# Exploring the sensory screening experiences of nurses working in long-term care homes with residents who have dementia: a qualitative study

**DOI:** 10.1186/s12877-018-0917-x

**Published:** 2018-10-04

**Authors:** Fiona Höbler, Xochil Argueta-Warden, Miriam Rodríguez-Monforte, Astrid Escrig-Pinol, Walter Wittich, Katherine S. McGilton

**Affiliations:** 1 Toronto Rehabilitation Institute-University Health Network, 130 Dunn Ave, Toronto, M6K 2R7 Canada; 2 Rehabilitation Sciences Institute, University of Toronto, Toronto, ON Canada; 3 Lawrence S. Bloomberg Faculty of Nursing, University of Toronto, 130 Dunn Ave, Toronto, M6K 2R7 Canada; 4 FCS Blanquerna-Universitat Ramon Llull, Carrer de Padilla, 326-332, 08025 Barcelona, Spain; 5 Dalla Lana School of Public Health, University of Toronto, Toronto, ON Canada; 6 School of Optometry, Université de Montréal, 3744, rue Jean-Brillant, 260-7, Montréal, Québec, H3T 1P1 Canada; 7 CRIR/Centre de réadaptation MAB-Mackay du CIUSSS du Centre-Ouest-de-l’Île-de-Montréal, Montréal, Québec, Canada; 8 CRIR/Institut Nazareth et Louis-Braille du CISSS de la Montérégie-Centre, Longueuil, Québec, Canada

**Keywords:** Nursing, Long-term care, Dementia, Visual impairment, Hearing loss, Sensory loss, Screening

## Abstract

**Background:**

The prevalence of vision and hearing loss is higher amongst older individuals with dementia, as well as higher in long-term care settings than in the wider community. However, the incidence of sensory impairment is underreported and often goes untreated. In this study, we aimed to understand nurses’ current experiences of screening and caring for long-term care residents who have dementia and sensory impairment.

**Methods:**

As part of a larger study on the sensory screening of long-term care residents with dementia, an environmental scan was conducted with front-line healthcare providers. We report here on the findings from the content analysis of individual, semi-structured interviews with nurses working in two long-term care homes in Southern Ontario, Canada. Twenty regulated nurses, including designated resident assessment coordinators, working full- or part-time with individuals who have dementia, participated across the two sites. All interviews were transcribed, and their contents reviewed and coded for themes by means of inductive thematic analysis.

**Results:**

Following a systematic and recursive approach, three analysts identified several themes relating to: 1) the sensory screening process, 2) communication strategies, and 3) quality of life, sensory loss, and dementia. Participants reported on the strengths and limitations of screening procedures, what improvements should be made, which informal strategies are effective, and the continued professional development that is needed.

**Conclusions:**

Nurses demonstrated insight into the facilitators and barriers to effective screening and care of residents with dementia and sensory impairments, and expressed the need for further education, more suitable screening tools, and formalised accountability within the screening process for vision and hearing loss in these long-term care residents.

**Electronic supplementary material:**

The online version of this article (10.1186/s12877-018-0917-x) contains supplementary material, which is available to authorized users.

## Background

Dementia is increasing in our aging population, and is directly reflected in growing incidence rates found in long-term care homes (LTCHs), as recorded in several countries worldwide. As LTCH residents tend to be older, they also tend to have higher levels and more severe physical and cognitive impairments than community-dwelling older adults [1]. The prevalence of dementia has been reported to be between 60 and 80% amongst residents in this setting [2, 3]. In addition, owing to its association with cognitive impairment, the prevalence of sensory impairment is also higher in this population [4]. More than 80% of residents have, at least, mild hearing loss and ∼50% a moderate to severe hearing impairment [5–8]. Impaired vision is twice as high amongst LTCH residents as in the general older population, estimated at 30% to 57% [9–12]. Whereas, hearing and/or vision loss can affect over 60% of LTCH residents; and estimates of combined vision and hearing impairment, known as dual sensory impairment (DSI), affects one third of LTCH residents [9]. Despite high prevalence rates, both dementia and sensory impairment are often undiagnosed, underreported or undocumented [3, 6, 13, 14].

Research of sensory screening measures in care settings, such as nursing homes [9], retirement homes [15], and LTCHs [16], has evidenced the need for assessment and intervention for this vulnerable population. In Canada and the United States, as in many countries across Europe and Australasia, the current standard for screening residents in these settings involves the Resident Assessment Instrument – Minimum Data Set 2.0 (RAI-MDS 2.0) [17]. This screening is advised to be carried out within two weeks of a resident’s admission to the LTCH by a regulated nurse, either a Registered Nurse (RN) or Registered Practical Nurse (RPN), and coordinated with the appropriate health professionals [18]. The screening process has the purpose of identifying individuals with a probability of having a disease or condition, so that they can be referred for appropriate diagnostic testing [19], as well as detecting a change in the resident’s status, to ensure their health, safety, and maximal functioning [20]. The RAI-MDS 2.0 comprises of subsections for vision and hearing, whereby data is gathered from observation, review of clinical records, care plans, and through interviews with the resident, as well as significant others and team members [21].

Assessment measures that rely on self-report or the observations of others have, however, been found to be less reliable [22–24] or accurate than standard objective techniques [25, 26]. Furthermore, individuals with dementia are often unable to complete hearing or vision screening self-report tests [27–29], due to communicative difficulties or problems understanding, and are less likely to be assessed than those without dementia [30]. This can create barriers to their access to necessary hearing and vision care from a specialist. Alongside residents with cognitive impairment, even LTCH residents with a single sensory impairment are more likely to be affected by communication problems, fatigue, balance problems, and sleep problems, than residents without impairment; with those who have dual impairment being at increased risk of faster cognitive decline [9].

With current methods of screening for sensory loss in LTCH residents reported as inadequate in sensitivity and appropriateness for older adults with dementia [31], a larger mixed-methods project was initiated to find the most effective vision and hearing screening tools suitable for this population [32]. As part of this investigation, an environmental scan was conducted with professionals working in hearing and vision care [33], as well as in nursing care, to identify tools, technologies, and strategies that are currently being used with clients who have dementia. Here, we report on the findings of our interviews with nurses who work with residents who have dementia in LTCHs, including nurses responsible for the sensory screening of these residents.

## Methods

This study was originally guided by the broader research question seeking to identify the most effective measures and strategies used by healthcare professionals to screen for hearing and vision loss in persons with dementia. During data collection, the use of such screening measures in LTCHs was found to be limited, thus the research question was adjusted to also explore the experiences of nurses in caring for and screening residents with dementia and sensory impairment living in LTCHs.

All participants attended semi-structured, in-person interviews, conducted on site with a clinically trained member of the research team (FH, MSc, female). Participating nurses were asked about: their experiences with persons who have dementia, as well as hearing and vision loss; how they identify which residents have impairments; ways in which assessments could be improved, and key elements to include in a sensory screening package. The questions and protocol used to guide the interview are outlined in the Interview Guide (see Additional file 1), to which a semi-structured approach was applied to allow for probing and further exploration of participants’ responses.

The study was conducted with the approval of the Research Ethics Boards of each LTC institution and in adherence to The Code of Ethics of the World Medical Association (Declaration of Helsinki) for experiments involving humans [34]. All participants provided informed written consent before participating in the study, and received compensation in-kind for their time and effort upon completion of their interview. The reporting of the study followed the published guidelines for qualitative research (see Additional file 2).

### Study site and participants

In-person interviews took place across two LTCHs in Southern Ontario, Canada, which ranged in scale between 100 and 450 residents in their care, respectively. The larger facility also accommodated on-site, specialist services in audiology and ophthalmology; whereas, the smaller of the two homes referred residents to off-site clinics. This was the primary difference between the LTCHs, as both were for-profit institutions, with equivalent assimilation and ratio of staffing. These staffing models involved one nursing assistant for every nine residents during the day, with supervision provided by charge nurses (RPN or RN). These two homes were selected for this research as they had dedicated units for the care of residents with dementia, whilst providing for contrasting models of care in how specialist health services were provided to the residents in their care.

The principal investigator of the study (KMG, PhD) made initial contact with the Assistant Directors of Nursing (ADON) at each LTCH to inform them about the study and its purpose. The ADONs first approached potential participants, based on the provided eligibility criteria. Eligible participants were registered nurses (RNs) and registered practical nurses (RPNs), working full- or part-time with residents who have dementia, and were responsible for administering hearing and/or vision screening tests to residents as part of their duties, in the form of the RAI-MDS or otherwise. Each LTCH had one designated member of nursing staff who held the position of RAI coordinator, and so participation was extended to registered nurses involved in other areas of care and assessment of residents with dementia. A purposive sample of 20 nurses was interviewed across the two LTCHs (*N* = 10 at each site). Sampling was based on the qualitative research standards reported for this type of exploratory research [35].

The participant characteristics are summarised in Table 1. Participants ranged in their years of experience of working in LTC (from 7 months to 30 years), working with residents who have dementia (from limited interaction to 22 years), as well as in their experience of assessing residents with dementia. The designate RAI-MDS coordinator at each site was included in the sample for analysis. The majority of participants were female, had college level education, were practising as registered practical nurses, and worked full-time.

**Table 1:** Summary of participant characteristics (*N* = 20)

Continuous Characteristics	Mean	[SD]
Years working in dementia care	9.63	[6.60]
Years working in LTC	10.48	[7.12]
Age [*N* = 18]	41.78	[7.52]
Categorical Characteristics	N	[%]
Registered Nurse	6	30
Employed full-time	14	70
Female	18	90

### Data collection

The semi-structured interviews were conducted in-person and on-site [36] between January and May 2016. Identified eligible participants were invited to an individual, confidential interview in a quiet and convenient location, and at a time of their choice. Participants were first individually briefed about the study, its purpose, the voluntary basis of their participation, and received answers to any questions or concerns they had. Written informed consent was obtained from all participants prior to the interview. The semi-structured protocol for the interviews allowed for questions to be asked as appropriate to the participating nurse’s role and experience, as well as to elaborate on information volunteered by the participant. Participants were encouraged to take adequate time to express their opinions, to do most of the talking, and advised that there was no right or wrong answer to any question. All interviews were audio-recorded, transcribed, proofed for completeness and accuracy, and anonymised prior to data analysis. Duration of interviews included in the analysis was a mean of 28 min 9 s [SD = 2.59].

### Data analysis

The data-driven analysis followed a thematic content approach as outlined by Graneheim and Lundman [37], and Braun and Clarke [38]. Table 2 outlines the methods employed for data analysis. The stepwise, recursive analysis began with three analysts (FH, MRM, XAW) familiarising themselves with the full data corpus (transcripts and audio recordings). Each of the 20 data sets were independently analysed by two members of the research team in the first level of coding [37]. The team met weekly to refine themes and to discuss disconfirming evidence emerging in the analysis. In this first level, the interview data were exhaustively coded into seven mutually exclusive themes [39]: LTC demographics, characteristics of participants, the process of care or screening, enablers of care, enablers of screening, barriers to care, and barriers to screening. Memos were made with analytical insights throughout this process.

**Table 2:** Phases of thematic analysis (adapted from Braun and Clarke, [38])

Phases of thematic analysis	Description of the process
1. Familiarization with the data	− The full corpus of interviews was transcribed and verified for accuracy. − The analysts first listened to, read and re-read all interviews to identify emerging trends and ideas in the data.
2. Generating initial codes	− Through group discussion and independent arbitration, the analysts agreed on initial codes: Barriers to Screening, Enablers of Screening, Barriers to Care, Enablers of Care, Process, LTC Demographics, and Participant Demographics. − The entire data set was systematically coded into exclusive categories of information. − Coding of data was reviewed by analysts and group consensus was reached. − Data was collated into each code manually.
3. Searching for themes	− The coded data were reviewed for common, emergent themes. − Thematic data was collated by each analyst manually.
4. Reviewing themes	− Themes were checked against all coded extracts, as well as against the entire data set for accuracy. − Identified themes were reviewed in group discussion with all three analysts and common links between the themes drawn.
5. Defining and naming themes	− Through recursive and independent review of full data sets, analysts verified the accuracy of the themes, and the overall story reported in the data. − Group discussion and a consensus approach was used for the generation of clear definitions and names for the main, overarching themes.
6. Producing the report	− Analysts selected representative and vivid extract examples to be included in the final report. − Final analysis of selected extracts was completed by relating the results of the analysis back to the research question and literature. − This scholarly report of the analysis was produced.

**Fig. 1:**
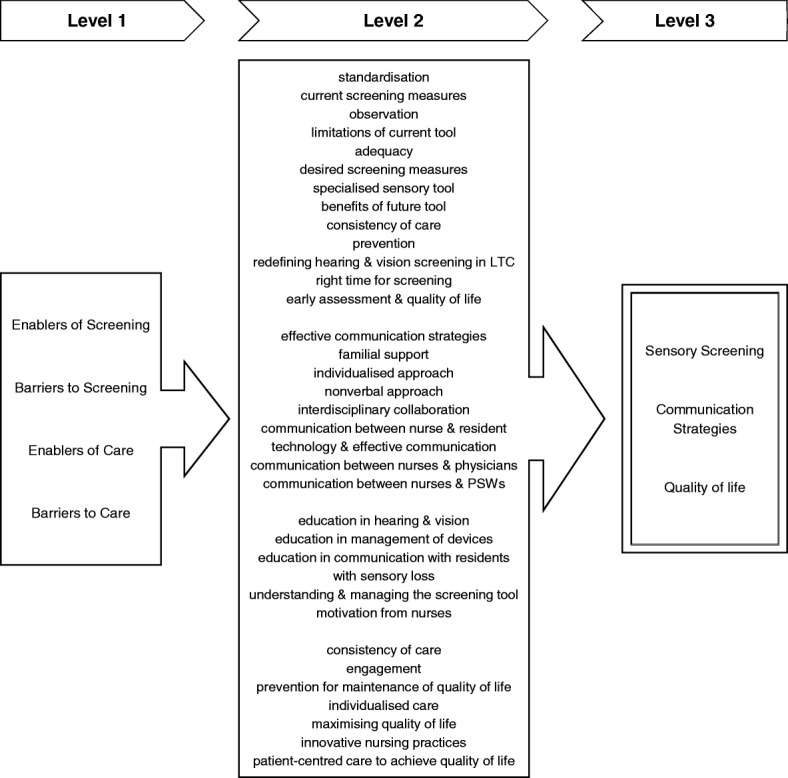
Schematic chart of thematic development. First level codes were developed into second level subthemes, which were then categorised into three main encompassing themes at the final stage of thematic analysis.

On the second level of analysis, subthemes within the data from the four main categories of interest (i.e. enablers of care, enablers of screening, barriers to care, and barriers to screening) were identified inductively. Data from the remaining three themes were used to inform the background of the study (i.e. LTCH demographics, characteristics of participants, the process of care or screening). The subthemes reflected the prominent themes that characterised the participant’s recommendations and concerns within the first level. These subthemes were then categorised into three main encompassing themes, which were used to present the results below (see Fig. 1 for Schematic chart of thematic development). Following the full content analysis, saturation of thematic data and supporting content information was assessed, which resulted in an agreed sample of the most richly informative citations, selected by the qualitative analysts, to represent the reported views and experiences of the interviewed participants [40, 41]

## Results

In discussing the barriers to and enablers of care and screening of long-term care residents with sensory loss and dementia, interviewed nurses provided insights into their experiences that were found to relate to three main themes. Interviewees described the current screening procedure across both sites in terms of its strengths and limitations, how screening measures should be more timely, suitable, and repeatable for residents with dementia, which informal screening strategies were being used instead, and what type of professional development was needed. Nurses also discussed how communication strategies were effectively used to circumvent gaps in the process, advocating for a person-centred approach and the effective application of interdisciplinary communication within the care team of the LTC setting. Ultimately, nurses highlighted that these implementations were essential for their quality of care and residents’ quality of life, as untreated sensory loss could lead to social isolation or responsive behaviours. They recommended the maintenance of quality of life and maximisation of sensory abilities through use of appropriate intervention and assistive technologies. The nurses’ comments on these topics are cited by reference to their LTC home (H#) and participant number (N#).

### The sensory screening process

Across both LTCHs, a similar process of screening by use of the RAI-MDS was employed. Each facility had a designate member of nursing staff who was responsible for screening residents upon admission, at regular intervals (quarterly and annually), and tracking when residents were due to be screened again, e.g., if the resident’s status changed. In the RAI-MDS, a section on “Hearing, Speech, and Vision” provides the examiner with two questions regarding the resident’s hearing ability and hearing aid use, along with two questions on visual ability and corrective lenses use. To complete these questions, the examiner is instructed to ask the resident about their hearing and visual function, as well as “the resident’s family, direct care staff, activities personnel,” and speech or hearing specialists in the case of hearing. Examiners are also instructed to observe the resident in their verbal interactions, and to use a book or newspaper with varying sizes of print to evaluate the respective functions [42]. If concerns related to vision and/or hearing are detected during screening, the resident is referred to the respective ophthalmologic, optometric and/or or audiologic specialist for diagnostic assessment.

The barriers to and enablers of this process were outlined by nurses in terms of the baseline information that was provided, the suitability of the measure and manner of testing for residents with dementia, as well as their own developed strategies for overcoming current barriers.

#### Strengths and limitations of current screening measures

The current screening measure in use was identical across both LTCHs, and nurses who were familiar with the RAI-MDS and its screening protocol recognised both the advantages and limitations of the tool. Although some participants referred to the benefits of its simplicity in providing a “baseline” [H2N8] or a “snapshot of the resident”[H1N3] and their overall health status, as well as “a little bit of a framework to operate in” [H2N8], the same nurses also indicated an interest in learning about alternative measures that could be more beneficial to assessment.

H1N2: “*It’s simple, and it works so far. So it’s kind of something that it’s handy”[…] “Maybe there’s other tools that they use out there […] so it’ll be interesting to learn or… It’ll be nice to know other useful tools that could be beneficial in doing some assessment*.”

Where both facilities differed was the availability of on-site audiology and ophthalmology clinics at the larger LTCH (H2), to which residents could be referred upon admission or due to a suspected change in their status. Nurses here acknowledged that this was unique to their facility and described the advantages of the accessibility of this service to residents and their families.

H2N9: “*We’re very fortunate here […] that everything is just in the facility. So, they don’t have to go out and families don’t really feel like ‘Oh, my gosh, I have to be there for this assessment’ […], then they’re more inclined to say okay, go ahead, do the assessment, whatever you want.*”

What was initially described as a snapshot of the resident’s profile could be enhanced with added baseline information through specialist input, provided in an efficient manner, which also benefited the nurses’ ability to care for their residents:

H2N9: “*And then once all the consults have been done, they’re usually done really quickly anyways, especially for new admissions, so that we can get a baseline. We know where they’re at, so we can care for them better.*”

#### A need for more timely, suitable, and repeatable screening

For other nurses, the information provided by current screening measures was seen as insufficient, and suggestions for improvement were made. Participants sought additional questions for screening, supplemental responses that could be accepted for non-verbal residents, and repeatable measures, so that the influences of a resident’s sensory and cognitive impairments could be distinguished.

H1N3: “*Oh, I love to change more. Put more questions in there. […] Like asking, okay, the resident has impaired cognition, has dementia, and then you show this pen and the resident cannot recognize it. What’s next? […] Because the resident can’t answer you accurately, […] I cannot say that oh, the resident has impaired vision. It doesn’t work that way. It should be like if there’s impaired cognition, what are these questions to be asked in order to determine the resident has vision impairment or hearing impairment.*”

Across both cohorts, differentiating between sensory and cognitive impairment in residents was reported as one of the nurses’ greatest challenges, which called the suitability of current measures into question.

H2N4: “*That’s actually one of the hardest assessments. […] I mean, there’s no way even for eyes and for hearing because they can’t, they can’t verbalize. That’s a challenge*”.

In addition to the need for tests to be better adapted to cognitively impaired individuals who are unable to provide a verbal response, several interviewees highlighted the importance of also considering the timing and repeatability of screening measures for these residents. These were also seen as not accommodated by the current screening schedule:

H1N9: “*You can’t just assess somebody one day and say, well, look, he can’t hear. You have to remember the status of the person” […] “So with dementia maybe they will hear me today, and then tomorrow they won’t. It depends on the person”; “[…] you don’t wait for three months and five months […] it takes a little bit for you to assess that person*.”

At the second LTCH, a nurse observed how repeated testing was implemented at their specialist clinics, and together with the knowledge of when the individual resident could perform at their best, a more reliable outcome could be measured.

H2N8: “*sometimes what they’ll do, they’ll have a recall, like another appointment another time to see if, okay, when they come and maybe they’ll be in a better mood when they come back, and they cooperate. […] But obviously you have to probably know the time of day that’s best for the client, too. […] You know, all these things you have to work in to get the most reliable tests in place, right*?”

Not only that the screening of residents should be scheduled at the optimal time of day for the individual, but nurses also highlighted the importance of early screening for timely detection of change or decline, calling for efficiencies in the screening and assessment process.

H2N8: “*You know, as I said it’s just the key element is timing, really; […] and making sure things are done in a certain amount of time in between so you catch, so things don’t deteriorate quickly on you.*”

Throughout the interviews, nurses revealed conscientiousness and insight into the reality of the residents’ well-being and experience of their cognitive status. Their developed familiarity with and sensitivity to the circumstances of residents in their care enabled them to understand the process from the individual’s perspective and fully account for the range of adaptations to be made.

H2N8: “*I think with dementia you have to kind of go into their own little world with them. The reality of what they’re feeling now is what they’re feeling now, right? You can’t really over-walk that reality, right? You have to go back, and they might feel one way this minute and the next minute they’re totally compliant with what you’re asking them to do. So when you get that compliant moment, then you go ahead with it, yeah*.”

#### Informal screening strategies

In the absence of suitable measures for residents with dementia, nurses described how they assumed the responsibility of further investigation, often by means of informal observation or self-developed strategies. One RAI Coordinator spoke of continuing the evaluation beyond that of the initial screening, to provide them with additional information on the resident’s functioning:

H1N3: “*It’s limited for only a few questions. It’s in general. So based on the general question we have to create our own assessment or questions to go in details.*”

Enriched by their experience, nurses used informal observation and their developed intuition to effectively and reliably screen their residents for possible sensory loss. In the following, nurses from both homes describe strategies for the evaluation residents’ hearing and vision, respectively:

H1N9: “*Because, remember, we are not here to diagnose anybody. Right? We’re here to help them. […] if I’m speaking to you right now and you can’t hear me, you would show me signs that you can’t hear me. You would say, “What did you say?” or you show me gesture, anything to … for me to indicate that you can’t hear me, right? […] And then I would probably do it a couple more times, not the same day; maybe the next day or two days after, whatever. And then, after that, then we can report that you can’t hear*.”

H2N2: “*It was just from experience. At least they know, they’re familiar with the letters, if you put their name on it […] they know. Like, if you […] put the random letters they not really might engage. But […] they know their names, they’re engaged in it. They will know*.”

As well as repeating their evaluations for a more reliable result, nurses’ strategies across both LTCHs involved the acceptance of meaningful responses and utilisation of meaningful stimuli, which were tailored by an individualised approach.

H2N4: “*For clients who have a certain stage of dementia; for end stage or who [don’t] verbalise, I don’t think that’s appropriate […].”*

H1N3: “*If it’s non-verbal resident because of dementia, there should be a non-verbal assessment for observation*.”

H1N2: “*You have to kind of tweak it or change it a bit on how you approach them. […] You don’t use the same approach on every […] resident*”.

#### A call for standardised practice and professional development

Despite the reported insights into informal assessment strategies that had been used effectively, the knowledge and experiences of sensory screening as described by nurses were variable. Few nurses were not aware of the RAI-MDS instrument or any type of screening process being available to them. Due to a lack of awareness regarding the standard protocol, nurses would also rely on their intuition.

H2N8: “*I’ve never worked anywhere where they really have any set formalities for nurses to check for hearing and vision, you know*”.

H2N4: *“…from my experience I’ve never conducted an official tool. Like it’s just basing on your nursing assessment, let’s say, the changes in behaviour, changes in the routine, but we don’t have the tool, let’s say, for the pain scale. We have a tool for skin assessment; we have a tool to use, but we don’t have for the hearing and for the visual*.”

However, with nurses’ experience and their developed insights varying across interviewees, they did share the concern that not all staff could adopt an observational approach and that, owing to a lack of consistency in knowledge and experience, there was indeed a need for standardisation of these screening approaches.

H2N8: “…*because some people are […] not observant, and people don’t carry out stuff, but when there’s a formal tool, then they have to be accountable for that formal tool, right*?”

Throughout the interviews, nurses expressed their desire to learn more about tools and strategies that can be used to better screen residents in their care. Nurses’ education was also described across both LTCHs as an area with potential for development and improvement, in order for the screening competencies of LTC staff, as well as the screening process as a whole, to be ameliorated. Nurses suggested approaches and areas of focus for this improvement.

H2N5: “*I think education sessions or, you know, trainings for nurses, get updates. Like I think those will be like really helpful*.”

H2N2: “*It can be improved, like, staff education … should be like tell staff how to, like, take care of the hearing aid better like handling wise, sometimes you have to teach them. […] some of them they bring new hearing aids from where we don’t know it’s - it’s high tech. So - but they always have better education is always good; like the staff needs to be just you know, reminded*.”

Education was reportedly provided by behavioural consultants to assist nurses in their understanding and management of responsive behaviours, which were recognised by one nurse participant as being impacted by underlying sensory impairments.

H2N4: “*Yeah, we do that and we’re still doing it; […] on how to speak with a client who has hearing impairment or vision impairment. […] one of them also is the behavioural consult to see if they can give the nurses or the PSW, you know, tools or pointers on how to react, how to respond, what to see, what to expect.”*

However, the knowledge and skill development required to carry out sensory screening was not reported to be currently offered or instructed through educational provision at either facility.

### Communication strategies

Throughout the interviews at each LTC home, communication was often cited as a key facilitator or barrier to the care and assessment of residents with dementia. A person-centred approach was seen as an enabler of informal assessment and of better care provision, along with effective interdisciplinary communication also contributing to both. When interdisciplinary communication broke down, however, this would present itself as a formidable barrier to the screening process.

#### Person-centred communication

As evidenced in the informal strategies adopted by nurses in a screening context, interviewed participants also advocated for person-centredness in their everyday communications with residents:

H1N9: “*it’s communication. That’s the key thing. You have to talk to the resident, depending on their status, so he will understand what you’re saying. […] It depends on the individual how much they could understand, and if you were interviewing them at the - for the first time you have different strategies or different things that you would do to make sure that that person understand what you’re saying. They have different barrier, like, language barrier. It depends on the individual […]”.*

When language or comprehension barriers existed, nurses described how going beyond verbal communication and using gesture was helpful in overcoming these and effectively connecting with their residents:

H2N9: “*communication is really hard, so you kind of use not just speech, but gestures and other tools that can help you communicate or get your, whatever you want to get across to them. More often than not, you really have to keep repeating yourself […] I’ll point to the ear, that this is for the ear, to help you. And then once it’s on, then you can see that there’s a change in their face and they start smiling and I guess, because they’re already hearing what you’re saying*.”

H2N8: “*If their vision and hearing is poor, maybe you can demonstrate what you need to do, right? And if they can’t see, it could be just taking their hands and putting the toothbrush in their hands and showing them that it’s a toothbrush, if they can understand.”*

Facilitative communication strategies consisted of simple language and environmental cues that helped engage residents who have dementia. At both sites, nurses shared their insights into using simple and effective communication strategies that were tailored to the individual.

H1N2: “*If they’re legally blind, obviously they can’t see you so you have to always have to introduce yourself to them and stuff. Even if they’re not blind […] you still have to introduce yourself.”*

H1N8: “*First of all, it is very effective to introduce yourself every now and then, because they have dementia so they forget who you are […] and then we using a simple words like can be answerable with yes or no*”.

H2N9: “…*like simple, direct words, will help. And if you say it in, I guess, a more clear, concise manner and if you notice that they’re sort of getting what you’re saying then the hearing is okay, but it could be that, you know, cognitive impairment is at play*”,

By these means, rapport with residents could be developed, as well as a better understanding of their cognitive abilities, and then a more valid assessment context could be enabled.

H1N2: “*I would say when someone has dementia and also have behaviour, you kind of have to talk to them first […] you have to gain their trust first. And then you do – like once they’re interactive and they’re talking to you and stuff […] That’s kind of a way of, I would say, doing the assessment as well*”.

H2N8: “*So it could be that, you know, they love a muffin. You know, you get a muffin and get a little coffee […] So you have to figure out how you’re going to approach somebody with this kind of stuff so you get the best possible result from them. So you can’t just go, ‘Okay, tell me what this means‘, you know*?”

Nurses’ communication strategies considered the person’s privacy, preferences, as well as impairments, and were individualised not only to the resident, but appropriate in their timing.

H1N8: “*Maybe you approach the resident in the wrong situation. Like maybe since lots of them they were incontinent, maybe something is bothering them like pain or sun-downing or they wanted to go to the washroom; so be sure before approaching the residents, you approach them on the right time*.”

#### The benefits of interdisciplinary communication

Beyond communicative interactions with the LTC residents, nurses placed an emphasis on the enablement provided by consistency of care and how this, in turn, facilitated effective interdisciplinary communication. The exchange of effective strategies and important considerations that are unique to the resident could help nurses learn how to overcome the barriers to communicating with and screening residents with more severe dementia.

H2N4: “*It’s, dementia - it’s very hard, especially if they’re not able to verbalise, if they don’t really know what’s happening to them. You really have to really get to know the clients. So therefore consistency is important for us […] because I don’t deal with the client, I only see them when I’ve been told by my PSW […] So if there’s a big change of staff, then there’s no way they would be able to see that there’s a change in that particular client. […] So I think caring for a client with dementia, consistency is one of the key rule that we are, for us to see and do a proper assessment*.”

The dependence upon the Personal Support Workers (PSWs), and their everyday familiarity with residents, was described as essential to the care and screening of residents by many nurses across both facilities. PSWs were highly regarded by nurses for having increased exposure to and heightened awareness of the residents who may experience a change of functional status.

H1N2: “*I don’t do hands on with each residents and stuff, so I rely to my staff like the PSW who is there every day taking care of them, doing care with them. So it’s just pretty much, I would say, communication has always had to be the key*.”

This communication was viewed as a reciprocal and collaborative effort to enable more reliable screening of residents. These efforts also extended to the larger care team to enable the identification of problems, as well as the most appropriate solutions.

H1N9: “…*I find that you need to work with your partner on the day shift or even the night shift. That’s how we work on this floor. If I’m not certain… because, as I said, we’re not here to diagnose. So I will communicate with my partner and say, “Well, this is what I feel.” And then, if she feels the same way, then we communicate together. Because, I said, I could be wrong, she could be wrong, so two heads is better than one*.”

H2N8: “*So we have that forum. So that’s why we have that status report that we meet with each other, we discuss issues and then we come up with solutions together. And then based on the solutions that we can use other team members, like other doctors, the social worker, other people get involved and help with solutions for the clients, you know*?”

This circle of care and collaboration was described as extending to the resident’s family, social workers, as well as additional medical and healthcare staff working with the resident, and provided for a multi-faceted team approach.

H2N8: “*all the facets are there, and then everybody has input and they bring up all these problems. It could be just – you know, the family will say, ‘I notice my mom is not seeing too well.’ It’s a good thing we have that family meeting because maybe we didn’t notice that. So then it comes to our attention and then based on the rounds, we come together as a team again and say well, maybe we should not only the ophthalmology, maybe we should get something else done or a screening test or – you know? So that’s how the support is there. You’re just not left alone to solve the problem.*”

#### Challenges to effective communication

A consistent and collaborative approach to care provision was seen as bridging the gap created by inexperience and unfamiliarity with the residents, as well as a valuable way of sharing information and strategies between staff on different schedules and different levels. However, communication was not always reported as effective and helpful. One participant described the sharing of information from ophthalmologists or audiologists to nurses at their facility as “not really communicated very well” [H2N5]. Assessment results and pertinent diagnoses were not always relayed to staff who worked closely with residents, and this information was sometimes difficult to retrieve.

H2N5: *“[…] once a referral is done or findings have been done I think it is also good for the nurses or the PSWs to know what’s really going on. […] Because sometimes that’s also missed, […] it goes like to the physician level already. […] Unless you go, you know, go deep to the transcription reports and to problem lists and all that and the notes. […] but then, you know, sometimes it’s written like not in a laymen’s term*.”

Even with procedures in place, there was still variability in how these were adhered to by members of the resident’s circle of care, and what was previously cited as enabling better screening became a barrier to the process instead:

H2N4: “*It can be improved. The reason why because sometimes there’s a little bit of a disconnect in the clinic. Because there are still some doctors […] I don’t think they are required to input their data in our electronic system. So they use the handwriting. […] sometimes it’s missed, what’s in there or they just write it in but they never tell us that there’s another follow-up, unless you really look and read it. So it’s still… it’s a work in progress*.”

Changes in status, in care and in diagnoses were seen as important points for communication amongst frontline healthcare providers, a process in which nurses assume a leading role. Nurse participants provided examples of how breakdowns in communication can be circumvented or improved upon to provide for better consistency and continuity of care, following the outcomes of assessment or its attempt.

H2N9: “*So our role as nurses is to communicate whatever we find, so that we can better care for residents, communicate it to the PSWs. So that is not only communicated verbally but it’s also done through care planning and whatnot, and we let them know that, you know, this is what the suggestion was, let’s try it, if it doesn’t work, at least we tried, do another strategy; […] The PSWs are very good. And they know their residents very well. Most of the times they would even suggest “Oh, let’s try this instead of that” to nurses or to doctors. And we steer the frontline, so we have to listen to them too*.”

### Quality of life, sensory loss, and dementia

The most oft-cited concern of nurses in relation to residents in their care who had vision or hearing loss, as well as cognitive impairment, was the impact of these challenges on their quality of life. Nurses identified the risks of social isolation and further decline in affected residents. They recognised the frustration caused by sensory impairment, and how this could lead to the incidence of responsive behaviours. They also confirmed the need for sensory screening, so that impairments can be detected early, and residents’ quality of life can thereby be maintained, as well as maximised, with appropriate interventions. The most effective, and often informal, means of doing so was through the provision of assistive devices.

#### Sensory loss and social isolation

Across the facilities, nurses recognised the impact of sensory loss on participation and engagement in LTCH communities, as well as its impact on the socialisation abilities and self-esteem of directly affected residents.

H1N8: “*You know, when you cannot hear anything you thought like everybody’s talking about you and you’re being isolated. […] one resident is deaf. Like she thought she’s being isolated or not being loved or appreciated. […] It will promote their self-esteem and feeling of being more confident.”*

H2N8: “*They tend to be a little bit more withdrawn. They’re not hearing anything, they’re probably not seeing anything. They may have the activity – they can’t participate, they’re just sitting there. To me it isolates them and it takes away from their quality of life, right, and engagement.”*

Sensory screening was seen as a means to providing residents with the support and devices they needed. One participant described the role of nurses in supporting and encouraging residents at the time of assessment as being facilitative to the process.

H1N8: “*First you have to comfort them, like “It’s okay; like if you are scared of going by yourself, I can accompany you.” Like spending quality time with them to give them emotional support and assurance*”.

#### Drawing the link between sensory loss and responsive behaviour

A link between sensory loss and responsive behaviour had previously been made by a participant on the subject of education; however, sensory screening for the purposes of early identification and appropriate intervention for such impairment was also noted as an important preventative factor of further decline and incidence of responsive behaviours:

H2N8: “*Maybe the person, they’re not hearing and they’re starting to get agitated, irritated. All these different, maybe responsive behaviours that come out of not seeing, not hearing, and then they don’t cooperate because it complicates the cognitive impairments more. And all that distress that they would’ve gone through would’ve been alleviated or stopped or – you know, prevented then, right?”*

To support and enable detection of impairments that may influence such behaviours, the right sensory screening measures were seen as essential. Nurses showed an appreciation for the impact of sensory loss on their residents and their concern for the frustration this can cause.

H2N4: “*So, I mean, in order for you to really get to know what’s causing the responsive behaviour, you need to have tools to do a proper assessment, right. Whether that could be a… let’s say hearing impairment; “I’m frustrated”, if you don’t hear you, get frustrated; and if you can’t see, you get frustrated. And like, is that one of the cause of their being aggressive or being frustrated would that be one? But it’s something that’s not actually… not seen though it’s part of the care but it’s not, you know, it’s not seen*.”

#### Maintaining and maximising quality of life

The nurses advocated for the maintenance of residents’ independence and quality of life, and saw the need to achieve this in the care setting, as well as at later stages of life. The nurses advocated for their residents’ right to access the activities that are enjoyable and stimulating to them.

H1N5: “*So they still have the rights to live, you know, as normal people. […] So those residents that likes to read, we’re still giving books to read, because ... in order that they enhance […] their brains more and then they can still function*”.

Participants described how both sensory impairment and dementia could negatively impact a resident’s quality of life, and identified the provision of routine, enabling participation and socialisation, and thereby maintaining independence and enjoyment, as significant mitigators:

H2N2: “…*if you take them out of their routine they get upset. You know. So or if their routine is […] since before they’re reading their newspaper, even though they don’t understand it, they know that they’ve been reading it, so if they - you don’t give it to them they get upset. So, then your day is - their day’s good, their day’s not good and your day’s not good*.”

H1N3: “*We do the assessments – we have to, the 24 hour care planning from main habits, what they do before coming in, what they do, what their job before. Because it will help us to give or like to maintain what the really social aspect of their life, right? When they were in the community. So at least we can provide those. Like, if the resident is fond of music, so we’re going to provide music. If the resident is dancing so we’re going to put into music program.”*

By detecting sensory loss through the initial screening, helpful diagnoses could be provided to the resident and family, as well as assistive intervention that enable residents to enjoy their daily activities at the LTCH.

H2N8: “*sometimes they find out that maybe they have glaucoma at that time, or maybe they really should have had glasses. Or maybe that’s why they were falling, because they probably weren’t seeing where they were going when they were at home, when the family wasn’t really noticing that that’s the reason why they were falling, you know? So that screening tool is very important. Then nursing on a whole, eventually after getting to know the clients we’ll be able to notice changes and be able to generate maybe other further assessments, you know, in between in case there are deteriorations*.”

Across both care homes, an appreciation of the significant contributions yielded by socialisation and sensory stimulation to residents was evident. Music programmes were reported as being especially impactful in providing positive engagement for residents with Alzheimer’s disease. This nurse described her observation and perceived importance of residents having access to music and sensory experiences.

H2N8: “*So once they can – especially people with Alzheimer’s, that music is so important. Music, and seeing colours and, you know, stuff like that. And so, if they can enjoy like even hearing music, it’s so important and they can sing and clap. You can see a joy come over them. They enjoy it, you know? So, and then even they even love to do the little exercise thing, and if they can see what she’s showing them, you know, it’s really important, at least you maximize […], or amplify whatever […] And it really helps. I’ve seen people improve on their socialization skills like that*.”

#### Assistive technologies as an important tool to enhance quality of life

Ultimately, assistive technology was deemed to be the greatest facilitator to residents with dementia and sensory loss engaging in activities and maintaining their quality of life at the LTCH. The successful outcome to sensory assessment was seen as the appropriate prescription of the most suitable assistive devices for the individual resident.

H1N3: “*So it’s either we get an upgrade eye glasses for the vision or we going to get a new or upgrade the hearing aid. So based on the referral. And then they will come back with the… whatever the recommendation is. […] Yeah, it’s effective because the resident can see more than the previous, or can hear more, or… Like we have one who has a hearing aid but then the hearing aid sometimes doesn’t work, so we change to pocket talker. So once the resident has a pocket talker she can respond more to the staff, can express more herself*.”

H2N8: “*I think it’s good. I’ve seen patients use it and it really helps. It really helps, it really helps, you know, with communication. They don’t seem so isolated and they obviously cannot hear from a low tone, a pocket talker then, and they’ll be involved in conversation and stuff like that*.”

In the absence of specialised technologies, alternative sensory aids were reportedly used across the facilities. For the hearing impaired, the pocket talker was widely seen as highly assistive; and for vision, a magnifying glass was commonly used instead of prescription lenses:

H2N8: “*Sometimes they use […] this big [magnifying glass]; and then for reading sometimes I see they have these big screens that they put a book under and they kind of amplify the words really bigger. I’ve seen residents enjoy that, but who likes – especially who likes to read before and then they can’t read anymore, that’s a good device to, um… especially for people with Alzheimer’s or cognitive impairment*.”

Such assistive devices were also found to be useful for testing purposes, and discriminating between sensory loss and other types of impairment.

H2N8: “*They may not sit long enough to do it but the amplifying glass, sometimes it’ll work, too. Or if you write words on a big paper, you know, to show them toothbrush so it’s big enough that you can see in case their vision is really off a bit*.”

## Discussion

The findings from this exploratory study demonstrate a recognition amongst nurses of the disconnect between current screening practices, with its limitations, and the complex reality for residents living with multiple impairments in LTCHs. In the absence of suitable screening tools or adaptable protocols, nurses provided insightful and instructive examples of how to overcome communication barriers or systemic constraints through person-centred and informal strategies in their care and assessment. Consistency of care and effective interdisciplinarity were found to be crucial to the success of these adapted procedures, but could not be relied upon under current circumstances, which brought participants back to the need for standardisation of and accountability for assessment practices.

The nurses in our study acknowledged, however, their wanting expertise in screening for hearing and vision loss; an area in which they have expressed a need and desire to be enabled by the provision of more effective screening tools that are suitable for individuals with dementia, and to be empowered to use these by means of more specialised professional development.

These results consolidate the findings from previous research, in which nurses have reported feeling inadequately trained to care for residents with sensory impairment [43], and frontline healthcare providers reported difficulties in distinguishing the relative contributions of sensory loss and dementia to breakdowns in communication [44, 45]. Despite the evidenced sensitivity and appropriateness of some sensory screening measures in older and non-communicative populations [27, 46–49], as well as the feasibility of in-service training for healthcare providers who are willing to engage in these [16], and its benefit to residents with cognitive and physical fragility [50], relevant evidence-based practice guidelines have not yet been adopted in these LTCH settings [51].

Even though the field of nursing has previously engaged in efforts to increase the awareness of sensory impairment in older adults in general, and has developed continuing education materials for this purpose [52, 53], nursing is not the only allied health profession that has encountered similar challenges where sensory abilities become relevant in health service delivery. Previous work in Canada, the US and the UK has highlighted the need for training on sensory screening and its rehabilitation by occupational therapists, and pointed out the absences of suitable training content on sensory abilities in educational curricula [54–57]. None of these efforts have yet advanced to a level where sensory and cognitive impairments are considered simultaneously during screening.

In this study, interviewed nurses notably acknowledged the relationship between hearing and vision, and quality of life (QoL), and how impairment in either can negatively influence the individual’s functioning as a whole, as well as their access to social engagement and participation. Research has shown that even without cognitive impairment, the ability to perform activities of daily living (ADLs), along with the QoL of LTCH residents, is significantly related to the presence of eye disorders or visual impairment [47, 58–61]. Likewise, access to social opportunities and QoL, is negatively impacted by hearing impairments [62, 63]. As suggested by nurses in the current study, the active management of dementia and coexisting conditions through coordination of care, as well as participation in activities and care programs [3], can improve QoL at all stages of severity for individuals directly affected, as well as for their care providers [64–66]. While ophthalmologist end otolaryngologists are often implicated in the medical sensory care of residents with dementia, their primary focus is on anatomy and physiology. Therefore, services provided by audiologists and optometrists that focus on functional ability are likely to be of great importance when considering the impact of sensory loss in this population, as well as being beneficial to the provision of specialised education to healthcare staff in this setting.

By use of a semi-structured interview approach in this study, respondents were provided with opportunity and encouragement to elaborate on responses, as well as to volunteer relevant information not directly related to questions [38]. Employing a qualitative content analysis of these interviews enabled a broad and reflexive search across the full data corpus for identification of the most prominent themes and meanings, which, in turn, informed and allowed for revision of the research question in line with the exploratory findings [67]. This study was limited to two LTC sites in Southern Ontario, Canada; however, these varied in their size and provision of services to residents. The investigation thereby captured ranging levels of exposure to assessment procedures, as well as varying levels of training and experience in caring for residents with dementia amongst participants. Despite differences in the provision of specialist healthcare services provided to residents by audiologists and ophthalmologists in the two LTCHs, regulated nurses reported common experiences and views regarding current standard procedures, as well as a shared need for additional education and the provision of more suitable sensory screening tools for residents.

In the current study, nurses highlighted communication as an important consideration in both the care and assessment of residents, providing insightful and creative examples of how they are personalising their approaches to communication, because many residents with sensory and cognitive impairment struggle most in this area. In addition, nurses recognised that hearing aids and glasses are not the sole solution to these problems. Through experience and team collaboration, successful communication strategies are shared and developed upon, and a patient-centred approach to care is at the core of their practice. Although communication strategies and strengths were identified as facilitative in overcoming the limitations of current screening procedures and interventions, this study did not explore the current procedures for assessing communication abilities. Future research is warranted in investigating the relative influence of communication, cognitive, and sensory abilities on interactions within the LTCH setting and on residents’ QoL, and which tools can enable nurses to more effectively identify relative impairments in LTC residents, which require appropriate intervention [68, 69].

## Conclusions

LTCH nurses demonstrated insight into the facilitators and barriers to effective screening and care of residents with dementia and possible vision and/or hearing impairments. Nurses recognised the limitations of current screening procedures, and emphasised the need for more suitable sensory assessment measures for residents with dementia, as well as their own expressed need for further education in carrying these out. Growing research supports the need for more appropriate screening measures and protocols to detect sensory decline in this vulnerable population, yet to be implemented in LTCHs. The current study, along with reports from previous research and governing bodies highlight the practicability and value of additional specialised training for registered nurses to empower these healthcare professionals to better screen, as well as feasibly diagnose and treat residents with complex needs under their care. The advancement of nurses’ roles and their potential to lead practice developments and service reform through staff education in managing sensory-related impairments and environmental modifications for residents has long been proposed [70–72]. To bridge the gap between such guidelines and current policies further curriculum development, continued professional education, as well as reinforcing on-site policies for training, knowledge translation and interdisciplinarity, is important for the enablement of nurses in the care and assessment of individuals with dementia and complex sensory needs, as underscored and called for by the nurses in this study.

## Additional files

Additional file 1: Interview guide, script and questions. This guide describes the responsibilities of the interviewer, the purpose of the interview, and outlines the interview questions. (DOCX 15 kb)

Additional file 2: Consolidated criteria for reporting qualitative studies (COREQ): 32-item checklist (adapted form Tong A, Sainsbury P, Craig J. 2007). This checklist provides the items included in the qualitative research report, and was adapted from Tong A, Sainsbury P, Craig J. Consolidated criteria for reporting qualitative research (COREQ): a 32-item checklist for interviews and focus groups. International journal for quality in health care. 2007;19(6), 349–357. (DOCX 16 kb)

## Abbreviations

ADON: Assistant Directors of Nursing
LTC: Long-term care
LTCH: Long-term care home
MDS: Minimum Data Set
PSW: Personal Support Worker
QoL: Quality of Life
RAI: Resident Assessment Instrument
REB: Research Ethics Board
RN: Registered Nurse
RPN: Registered Practical Nurse
