# Supplementary material for: Exploring the sensory screening experiences of nurses working in long-term care homes with residents who have dementia: a qualitative study
Source: BMC Geriatr. 2018 Oct 4;18:235. doi: 10.1186/s12877-018-0917-x (PMC6172849; doi:10.1186/s12877-018-0917-x)
Supplement: Supplementary file 1 — Interview guide, script and questions. This guide describes the responsibilities of the interviewer, the purpose of the interview, and outlines the interview questions. (DOCX 15 kb) [file 12877_2018_917_MOESM1_ESM.docx]

**Interview Guide**

The interview is conducted by the research analyst, whose responsibilities include:

- Use of a prepared script (below) to welcome participant and remind them of the purpose of the interview.
- Prior to beginning the interview, confirm with participant that all their questions regarding the study were answered.
- Ask the questions in the interview guide as applicable; may paraphrase to clarify what participants are saying.
- Adequately cover all applicable questions within the time allotted.
- Encourage all participants to talk and fully explain their answers (e.g. “Can you talk about that more?” “Help me understand what you mean.” “Can you give an example?”)
- Use of the audio recorder.
- Take detailed notes, in case the recorder fails or recording is inaudible, and to supplement analysis.
- Note/record subtle but relevant behaviours/ emotions that may not be captured in a quote e.g. tone.

It is good moderator practice to paraphrase and summarize long, complex or ambiguous comments. It demonstrates active listening and clarifies the comment for everyone in the group.

Ending the interview:

When the interview is complete the moderator thanks the participant.

**Interview Script**

Welcome:

Thanks for agreeing to be part of this interview. We appreciate your willingness to participate.

Introductions:

Moderator (possibly assistant moderator)

Purpose of Interview:

The reason we are having these interviews is to find out about your thoughts and experiences of using tools, technologies, and strategies to screen for vision and hearing impairment in persons who have dementia at your facility.

We have also completed a comprehensive literature review on screening tools, technologies, and strategies that are available and we now need your feedback to find out which and how such tools, technologies, and strategies are used in Long-Term Care facilities in Ontario.

The information gathered from these interviews will also inform the selection of sensory screening tools and scoring procedures which will be developed by the research team in consultation with a panel of content experts.

Our main objective is to identify effective vision and screening tools suitable for older adults with dementia, and adapt their administration if necessary. These tools will facilitate identifying those in need of referral to a vision or hearing professional. We hope that through individually tailored care for residents, the health and well-being of older adults with dementia will be promoted by enabling this population to participate more fully in programs and care activities offered in LTC.

Rules to make clear prior to the interview:

1. We want you to do the talking.

We are looking for rich data that typically is gathered when the participant has adequate time to express their opinions. As such, it is likely the moderators will be silent between questions.

1. There are no right or wrong answers.

Every person's experiences and opinions are important. We want to hear a wide range of opinions.

1. We will be audio recording the interview.

We want to capture everything you have to say.

**Interview Questions**

- Do you administer hearing and vision screening tests to residents at this facility?
- How long have you been working with persons who have dementia?
- Please describe your experience of working with persons who have dementia, as well as persons with hearing and vision loss.
- How do you differentiate between residents who have difficulty understanding due to a hearing impairment and those with cognitive impairments?
- How are residents identified as being in need of assessment for vision and hearing impairment?
  - Is there a standard procedure in place, and, if so, what are the steps?
  - What is the procedure for referring residents to a hearing and/or vision specialist?
- What tools, technologies, and strategies are you currently using to assess and/or detect vision and/or hearing in persons who have dementia?
- What approach is used or do you use in assessing persons who have dementia, e.g. what combination of tools, technologies, and strategies is being implemented?
- How do these tools, technologies, and strategies identify a person as having hearing and/or vision impairments?
- What are the facilitators and barriers in implementing this approach and/or procedure?
  - Please list all the challenges in using these tools, technologies, and strategies.
  - Please also list all the benefits to using these tools, technologies, and strategies.
- In what way can these tools, technologies, and strategies be improved?
- In your opinion, what are the key elements to include in a hearing and vision screening package for persons who have dementia?
- Do you have knowledge of or experience in using either of the following:
  - **Peek** application (turns a smartphone into an eye exam tool)?

…or similar other apps?

- - **uHear** application (downloadable audiometer on to an iPod Touch as a test for hearing loss)?

…or similar other apps?
