# Supplementary material for: Exploring the sensory screening experiences of nurses working in long-term care homes with residents who have dementia: a qualitative study
Source: BMC Geriatr. 2018 Oct 4;18:235. doi: 10.1186/s12877-018-0917-x (PMC6172849; doi:10.1186/s12877-018-0917-x)
Supplement: Supplementary file 2 — Consolidated criteria for reporting qualitative studies (COREQ): 32-item checklist (adapted form Tong A, Sainsbury P, Craig J. 2007). This checklist provides the items included in the qualitative research report, and was adapted from Tong A, Sainsbury P, Craig J. Consolidated criteria for reporting qualitative research (COREQ): a 32-item checklist for interviews and focus groups. International journal for quality in health care. 2007;19(6), 349–357. (DOCX 16 kb) [file 12877_2018_917_MOESM2_ESM.docx]

| **Topic / Item #** | **Guide questions/description** | **Reported on Page #** |
| --- | --- | --- |
| **Domain 1: Research team and reﬂexivity** |  |  |
| *Personal Characteristics* |  |  |
| 1. Interviewer/facilitator | Which author/s conducted the interview or focus group? | FH; Page 5 |
| 2. Credentials | What were the researcher’s credentials? E.g. PhD, MD | FH, MSc; Page 5  KMG, PhD; Page 6 |
| 3. Occupation | What was their occupation at the time of the study? | FH, Clinical Research Coordinator; Page 5  KMG, Primary Investigator; Page 6 |
| 4. Gender | Was the researcher male or female? | FH, female; Page 6 |
| 5. Experience and training | What experience or training did the researcher have? | FH, Clinical training; Page 6 |
| *Relationship with participants* |  |  |
| 6. Relationship established | Was a relationship established prior to study commencement? | No previous relationship to report. |
| 7. Participant knowledge of the interviewer | What did the participants know about the researcher? e.g. personal goals, reasons for doing the research | Reasons for the research explained as part of the consent process. |
| 8. Interviewer characteristics | What characteristics were reported about the inter viewer/facilitator? e.g. Bias, assumptions, reasons and interests in the research topic | No bias or assumptions to report. |
| **Domain 2: study design** |  |  |
| *Theoretical framework* |  |  |
| 9. Methodological orientation and Theory | What methodological orientation was stated to underpin the study? e.g. grounded theory, discourse analysis, ethnography, phenomenology, content analysis | Thematic content analysis; page 7. |
| *Participant selection* |  |  |
| 10. Sampling | How were participants selected? e.g. purposive, convenience, consecutive, snowball | Purposive sampling;  page 6. |
| 11. Method of approach | How were participants approached? e.g. face-to-face, telephone, mail, email | In-person; page 6. |
| 12. Sample size | How many participants were in the study? | N=20; page 6, Table 1. |
| 13. Non-participation | How many people refused to participate or dropped out? Reasons? | No one refused to participate. |
| *Setting* |  |  |
| 14. Setting of data collection | Where was the data collected? e.g. home, clinic, workplace | Workplace/on-site, page 7. |
| 15. Presence of non-participants | Was anyone else present besides the participants and researchers? | No one else was present. |
| 16. Description of sample | What are the important characteristics of the sample? e.g. demographic data, date | Demographic data, and years’ experience working in dementia care/LTC; Table 1. |
| *Data collection* |  |  |
| 17. Interview guide | Were questions, prompts, guides provided by the authors? Was it pilot tested? | Additional File 1: Interview Guide. |
| 18. Repeat interviews | Were repeat interviews carried out? If yes, how many? | No repeat interviews were carried out. |
| 19. Audio/visual recording | Did the research use audio or visual recording to collect the data? | Audio recording; page 7. |
| 20. Field notes | Were ﬁeld notes made during and/or after the interview or focus group? | Memos were made after the interview and during data analysis. |
| 21. Duration | What was the duration of the interviews or focus group? | Mean duration of 28 minutes 9 seconds [SD=2.59]; page 7. |
| 22. Data saturation | Was data saturation discussed? | Yes, on page 8. |
| 23. Transcripts returned | Were transcripts returned to participants for comment and/or correction? | No transcripts were returned. |
| **Domain 3: analysis and ﬁndings** |  |  |
| *Data analysis* |  |  |
| 24. Number of data coders | How many data coders coded the data? | Three coders: FH, MRM, XAW; page 7. |
| 25. Description of the coding tree | Did authors provide a description of the coding tree? | Data Analysis is described on pages 7-8. The coding tree is outlined in “Table 2: Phases of Thematic Analysis”, and “Figure 1: Schematic chart of thematic development”. |
| 26. Derivation of themes | Were themes identiﬁed in advance or derived from the data? | Identified themes were derived from the data; page 7-8, Table 2, Figure 1. |
| 27. Software | What software, if applicable, was used to manage the data? | No software was used to manage the data. Interview data were transcribed to Microsoft Word documents and codes were developed on Microsoft Excel sheets. |
| 28. Participant checking | Did participants provide feedback on the ﬁndings? | Participants were not asked to provide feedback on the ﬁndings. |
| *Reporting* |  |  |
| 29. Quotations presented | Were participant quotations presented to illustrate the themes/ﬁndings? Was each quotation identiﬁed? e.g. participant number | Participant quotations were presented to illustrate the themes and ﬁndings only identiﬁed by participant number and site number; in order to facilitate the anonymity of participants; pages 9-26. |
| 30. Data and ﬁndings consistent | Was there consistency between the data presented and the ﬁndings? | The data presented and the ﬁndings are consistent with previous research; pages 26-29. |
| 31. Clarity of major themes | Were major themes clearly presented in the ﬁndings? | Themes are presented in the Results section, pages 9-26. |
| 32. Clarity of minor themes | Is there a description of diverse cases or discussion of minor themes? | Any minor themes are discussed throughout the Results and Discussion sections; pages 9-29. |

**Reference:** Tong A, Sainsbury P, Craig J. Consolidated criteria for reporting qualitative research (COREQ): a 32-item checklist for interviews and focus groups. International journal for quality in health care. 2007;19(6), 349-357.
